# Toward the identification of molecular markers associated with phytochemical traits in the Iranian sumac (*Rhus coriaria* L.) population

**DOI:** 10.1002/fsn3.2273

**Published:** 2021-05-03

**Authors:** Rasoul Mohammadi Alaghuz, Reza Darvishzadeh, Ahmad Alijanpour, Mitra Razi

**Affiliations:** ^1^ Agricultural Biotechnology Department of Plant Production and Genetics Faculty of Agriculture and Natural Resources Urmia University Urmia Iran; ^2^ Department of Plant Production and Genetics Faculty of Agriculture and Natural Resources Urmia University Urmia Iran; ^3^ Department of Forestry Faculty of Agriculture and Natural Resources Urmia University Urmia Iran; ^4^ Department of Plant Production and Genetics Faculty of Agriculture and Natural Resources Urmia University Urmia Iran

**Keywords:** Association analysis, forest tree breeding, ISSR, linkage disequilibrium, phytochemical component, *Rhus coriaria*

## Abstract

Sumac (*Rhus coriaria* L.) is one of the important forest species dispersed in the northwest of Iran. It is one of the important spice in Iran and the Middle East because of active components containing organic acids, phenolic acids, flavonoids, anthocyanins, tannins and terpenoids. This study aimed to investigate population structure and linkage disequilibrium (LD) extent within *Rhus coriaria* L. genotypes using ISSR markers and identify molecular markers associated with phytochemical traits using association analysis. In the molecular part of the experiment, the genetic diversity of 75 sumac genotypes from five different areas of northwest Iran was assessed by 18 ISSR primers. In the phenotypic assessment, the fruits of the sumac genotypes were analyzed using HPLC‐LC/MSMS for determining phytochemical components including maleic acid, ellagic acid, maleic acid hexoside, gallic acid, coumaric acid, quercetin, caftaric acid, and linoleic acid. The phenotypic data analysis revealed the great phenotypic diversity among and within Iranian sumac populations for the studied phytochemical traits. The studied sumac genotypes were divided into two subpopulations based on molecular marker‐based structure analysis. A significant level of LD was observed in 11.64% of the ISSR marker pairs (*p* < .05). The mixed linear model procedure showed that 12 loci had a significant association with investigated traits. The ISSR loci identified in this study can be potentially used in marker‐assisted selection in sumac breeding programs.

## Graphical abstract

Present work was conducted to investigate population structure in a collection of Iranian sumac germplasm and identify the association of ISSR markers with phytochemical traits in the plant. A total 12 ISSR markers associated with studied traits was identified using an association mapping approach. These ISSR markers provide primary molecular information for marker‐assisted selection in sumac.

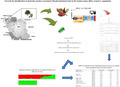

## 1 INTRODUCTION

Sumac (*Rhus coriaria* L.) belongs to the Anacardiaceae family and includes over 200 species throughout the world (Giovanelli et al., 2017; Mozaffarian, 2013; Peter, 2012). *R. coriaria* is the only species growing in Iran. It is a small tree with brown to red fruits, from which the spice is derived (Mozaffarian, 2013; Peter, 2012; Shabbir, 2012). Sumac is used in pure form or combination with other spices (Ali‐Shtayeh & Jamous, 2008). It has been used in traditional medicine for centuries to treat diseases such as cancer, diarrhea, hematemesis, diabetes, teeth and gum ailments, headaches, and stomach ache (Ali‐Shtayeh & Jamous, 2008; Shafiei et al., 2011).

Plants have different classes of phytochemicals. Phenolic compounds and flavonoids are the largest phytochemical molecules with antioxidant properties in plants (Andreu et al., 2018; Okpuzor et al., 2009; Ryu et al., 2006; Wang et al., 2016; Zahoor et al., 2018). Many research works showed that medicinal plants, fruits, and vegetables have various proteins, enzymes, and phytochemicals with antioxidant activity, which are the reason for their useful health effects (Scalbert et al., 2005; Zhang et al., 2016). A few studies were performed on determining the chemical composition of *R. coriaria* leaves and fruit epicarps (Regazzoni et al., 2013; Van Loo et al., 1988). *R. coriaria* has a wide range of active components containing hydrolysable tannins and various organic acids such as malic and citric acids (Kossah et al., 2010; Shabbir, 2012), fatty acids, vitamins, and terpenoid derivatives (Abu‐Reidah et al., 2014). Phenotyping of complex metabolomic characteristics is done easily by the metabolomics tools, such as gas chromatography–mass spectrometer (GC‐MS) (Saito & Matsuda, 2010).

Acquisition of any detailed information on genetic controlling of phenotypic variations is an important step before running any breeding activity in plants including sumac (Jin et al., 2010). The development of molecular marker technology has facilitated the identification of the genetic basis of quantitative traits (Semagn et al., 2010). ISSR is a microsatellite‐based method that does not require basic information on the genome and design of primers and produces many polymorphic patterns (Zietkiewicz et al., 1994). This marker is widely used in studies of genetic diversity, phylogeny, genomic mapping, and evolutionary biology in a wide range of crop and medicinal plant species (Reddy et al., 2002). Linkage and association analyses are the two most used methods for elucidating the genetic structure of quantitative or metric traits in plants. Association study was first applied in 2001 in maize concerning mapping quantitative trait loci (QTL) controlling flowering time (Thornsberry et al., 2001). Association analysis identifies the genotypic markers associated with variation of phenotypic traits based on “naturally produced linkage disequilibrium (LD),” and it has higher resolution (Flint‐Garcia et al., 2005; Xu et al., 2020) and takes shorter research time than linkage analysis (Flint‐Garcia et al., 2003). For running effective association analysis, LD should be surveyed in each region of chromosome or linkage group (Abdurakhmonov et al., 2008; Sorkheh et al., 2008). Some factors like small population size, inbreeding, population admixture, genetic drift, autogamy and epistasis increase LD, vice versa, allogamy, and high recombination and mutation rate decrease LD level (Al‐Maskri et al., 2012). Patterns of LD have been investigated in several crop species. It was observed, LD decays rapidly in some plants, for instance, within 1.1 kb in cultivated sunflower genotypes (Liu & Burke, 2006) or 300 bp in wild grapevine (Lijavetzky et al., 2007), whereas it decays slowly in some plants, for instance, within 100–200 kb in rice diverse lines (Huang et al., 2010; McNally et al., 2009) or 250 kb in cultivated soybean genotypes (Lam et al., 2010). In association analysis studies, the true association between a genotypic marker and QTL is a point of considerable interest. The existence of genotypes from different origins within the population (association panel) results in LD between unlinked loci. On other hand, the amount of LD in an association panel is associated with effective population size (Ne) (Sved, 1971); normally in a population with small size, the higher relatedness among genotypes (kinship) is seen so that it results in higher LD estimation (Falconer & Mackay, 1996). Then, structured population and kinship result in biased estimation of LD, which may augment the rate of false positives in association studies, that is, identifying markers that are not being involved in the variation of quantitative traits (Bradbury et al., 2007).

There are some statistical analyses for preventing population structure and kinship effects on association analysis (Cardon & Palme, 2003). A mixed linear model (MLM) is one of the statistical approaches that minimize the effect of troublesome factors such as population structure and kinship by incorporating these effects as covariates in the model (Yu et al., 2006). AM has been used in maize, wheat, rice, barley, and sorghum to clarify the genetic structure of complex traits such as yield, flowering, and disease resistance (Agrama et al., 2007; Breseghello & Sorrells, 2006; Cockram et al., 2010; Maccaferri et al., 2011; Murray et al., 2009; Ravel et al., 2006; Stracke et al., 2009; Thornsberry et al., 2001; Tommasini et al., 2007; Zhang et al., 2016). AM has also been used in forest trees and fruit crops like conifers (Eckert et al., 2010; Gonzalez‐Martinez et al., 2007), eucalyptus (Thumma et al., 2005), peach (Aranzana et al., 2010; Cevik et al., 2009), and grape (Chitwood et al., 2014; Emanuelli et al., 2010; Tello et al., 2016) for QTL mapping. The objectives of the present study were to investigate population structure and LD extent within *Rhus coriaria* L. population using ISSR markers and identify molecular markers associated with phytochemical traits using association analysis.

## 2 MATERIALS AND METHODS

### 2.1 Plant material

**FIGURE 1:**
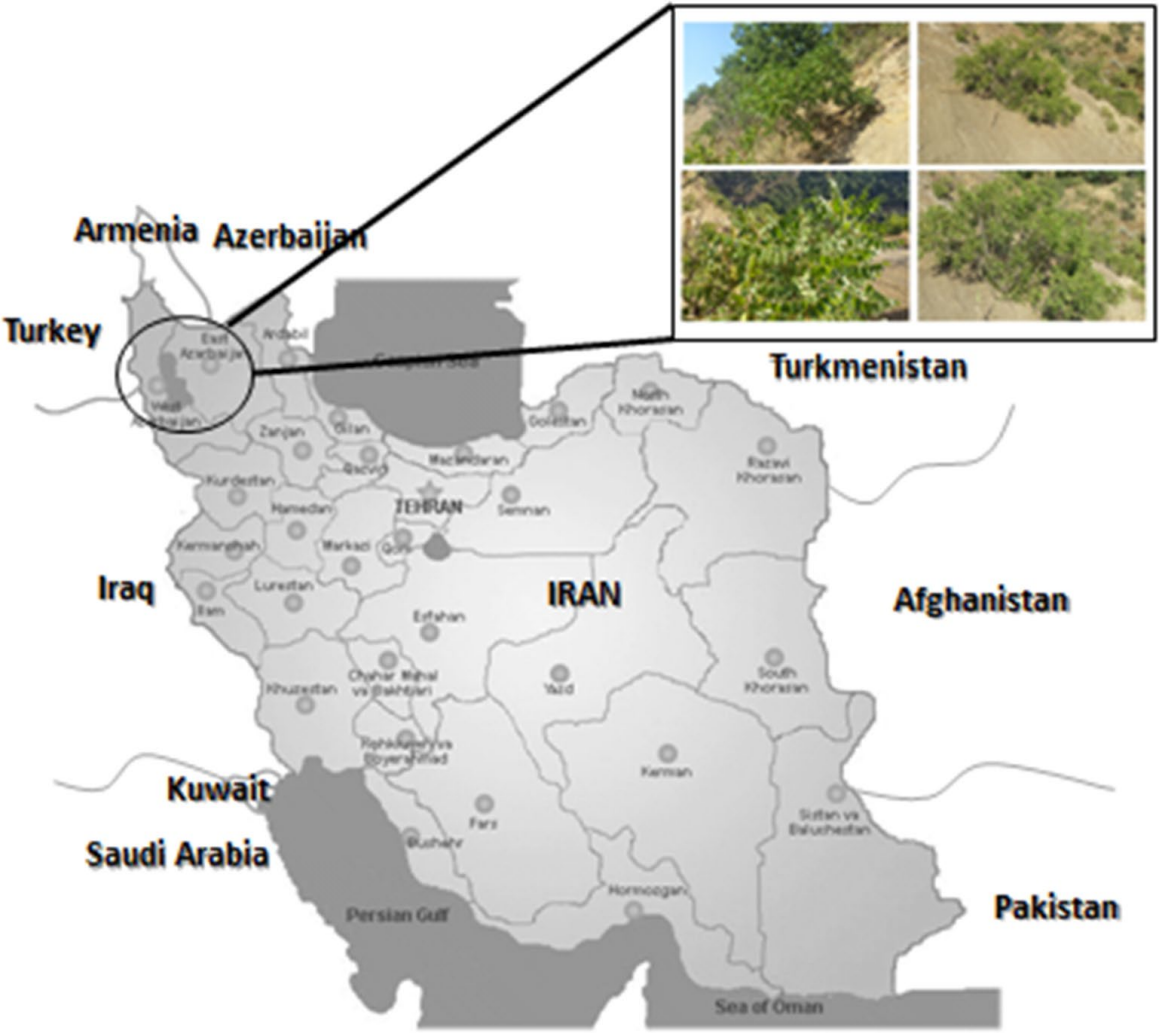
Regions where sumac populations were sampled

Seventy‐five sumac genotypes from five different regions (Kachleh, Dareh Khan and Dareh Nizh in West Azerbaijan and Horand‐Aghberaz and Vinagh in East Azerbaijan) of Iran were sampled (Figure 1, Table 1).

**TABLE 1:** Geographical location of the sumac fruit collection sites and selected ISSR primers for evaluation of sumac genetic diversity

Information of studied population				
Site	Population	latitude	Longitude	Height (m)
1	West Azerbaijan (Kachleh)	37º12′	44º52′	1727
2	West Azerbaijan (Dareh‐Khan)	37º18′	45º60′	1533
3	West Azerbaijan (Dareh‐Nezh)	37º16′	45º80′	1623
4	East Azerbaijan (Aghberaz)	38º59′	47º23′	1,190
5	East Azerbaijan (Vinagh)	39º 02′	46º50′	860

Information on ISSR primers				
Number	Primer	Repeated motif	5` to 3` sequence of primer	Annealing temperature
1	UBC809	(AG)8G	AGAGAGAGAGAGAGAGG	46
2	UBC810	(GA)8T	GAG AGA GAG AGA GAG AT	38
3	UBC811	(GA)8C	GAG AGA GAG AGA GAG AC	49
4	UBC816	(GA)8T	CAC ACA CAC ACA CAC AT	50
5	UBC823	(TC)8C	TCT CTC TCT CTC TCT CC	48
6	UBC826	(AC)8C	ACA CAC ACA CAC ACA CC	50
7	UBC834	(GA)8YT	AGA GAG AGA GAG AGA GYT	50
8	UBC835	(AG)8YC	AGA GAG AGA GAG AGA GYC	52
9	UBC841	(GA)8CTC	GAG AGA GAG AGA GAG AYC	52
10	UBC842	(GA)8CTG	GAG AGA GAG AGA GAG AYG	50
11	UBC854	(TC)8RG	TCT CTC TCT CTC TCT CRG	44
12	UBC856	(AC)8YA	ACA CAC ACA CAC ACA CYA	44
13	UBC857	(AC)8YG	ACA CAC ACA CAC ACA CYG	40
14	UBC864	(ATG)6	ATG ATG ATG ATG ATG ATG	42
15	UBC866	(CTC)6	CTC CTC CTC CTC CTC CTC	53
16	UBC867	(GGC)8	GGC GGC GGC GGC GGC GGC	39
17	UBC890	VHV (GT)7	VHV GTG TGT GTG TGT GT	52
18	UBC801	(AT)8T	ATA TAT ATA TAT ATA TT	‐

**TABLE** **1** Geographical location of the sumac fruit collection sites and selected ISSR primers for evaluation of sumac genetic diversity.

### 2.2 Evaluation of Phytochemical traits

#### 2.2.1 Sample preparation

Fruits of sumac were collected and dried. Epicarps of fruits were separated from kernels and ground to powder by the household mill. The powder was kept at room temperature until extraction.

#### 2.2.2 Extraction of phenolic compounds

Ten milliliters of methanol (HPLC grade) (80% v/v) added to grounded fruit epicarps (0.5 gr) and sonicated for 45 min at room temperature. The mixture was centrifuged for 15 min at 3,000 g at room temperature and then filtered through a 0.2 µm syringe filter (PALL, USA) and stored at 20ºC until analysis.

#### 2.2.3 HPLC‐LC/MS‐MS analysis

**FIGURE 2:**
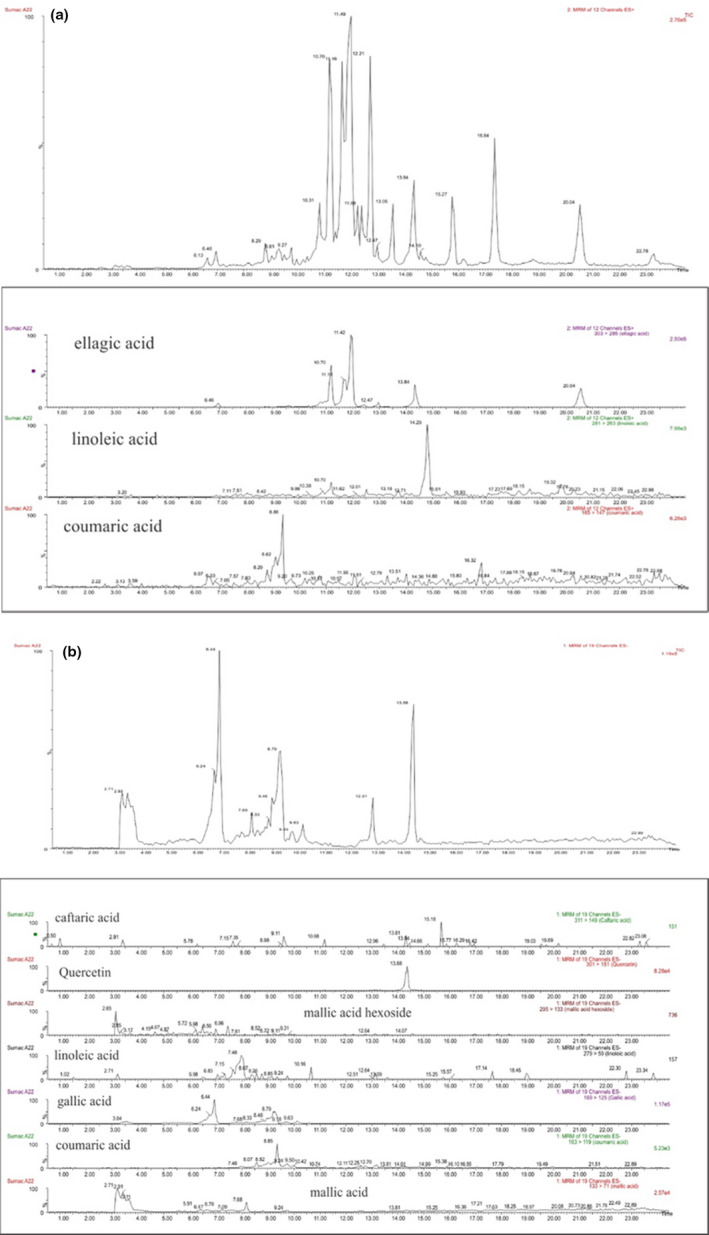
Detection of Iranian sumac compound. Analysis was performed in positive and negative ion mode. A, Standard chromatogram in positive electrospray (ESI⁺). B, Standard chromatogram in negative electrospray (ESI⁻)

Chromatographic separation was performed using an Agilent ZORBAX SB‐C18 (4.6 × 150 mm, 5 µm). Mass Lynx software, version 4.1, was used for instrument control and data acquisition. The analysis was performed in positive and negative ion mode (Figure 2).

### 2.3 ISSR genotyping

Genomic DNA extraction was performed following the method described by Doyle and Doyle (1990). Eighteen ISSR primers were used for genotyping of individuals (Table 1). The PCR program consisted of an initial denaturation at 94°C for 4 m, followed by 38 cycles of 94°C for 30 s, annealing temperature depending on primers composition (52 to 60ºC, Table 1) for 30 s, and 72°C for 2 m, with a final extension at 72°C for 10 m. Scoring of separated amplified fragments in agarose gels was performed according to present (1) or absence (0) of bands, and the produced binary data were used for statistical analysis.

### 2.4 Data analysis

In order to clarify the phenotypic variation within and among populations, the descriptive statistic was calculated using SAS software version 9.4. Estimating the population structure was performed using the Bayesian approach in the software package Structure 2.3.4 (Pritchard et al., 2000). Ten independent runs were applied. Five replications were considered for each run. The runs were performed under correlated allele frequencies and the admixture model. The burn‐in period length was 100,000, followed by 100,000 MCMC (Markov Chain Monte Carlo) replications. The optimal subpopulations number (K) was chosen considering the log probability of data [LnP(D)] (Rosenberg et al., 2002) and ΔK method presented by Evanno et al., (2005). Individuals with membership probability of equal or greater than 0.70 were allocated to subpopulations, while those with lower probabilities were assigned as admixed. Q‐matrix was derived from the last run of Structure (Pritchard et al., 2000). The linkage disequilibrium (LD) was calculated with TASSEL 2.1 (Bradbury et al., 2007). Association analysis was performed to analyze marker–trait association using a mixed linear model (MLM) including both Q and K matrices (Yu et al., 2006) as covariates in the model using TASSEL software.

## 3 RESULTS

### 3.1 Phenotypic variability

The descriptive statistics including central tendency and variability for studied traits were calculated and summarized in Table 2. A high level of phenotypic variability was observed among and within populations for studied traits in sumac populations (Table 2). The highest (708.01) and lowest (355.52) mean value for gallic acid 6.5 was observed in population 4 (Aghberaz; East Azerbaijan) and population 2 (Dareh‐Khan; West Azerbaijan), respectively. The highest (887) and lowest (532.54) mean value for gallic acid 8.7 was observed in population 2 (Dareh‐Khan; West Azerbaijan) and population 5 (Vinagh; East Azerbaijan). Concerning quercetin, the highest (92.29) and lowest (42.82) mean value was observed in population 1 (Kachleh; West Azerbaijan) and population 4 (Aghberaz; East Azerbaijan), respectively. About the malic acid, the highest (77,321.64) and lowest (7,393.5) mean value was observed in population 2 (Dareh‐Khan; West Azerbaijan) and population 5 (Vinagh; East Azerbaijan), respectively. The highest (1940.35) and lowest (525.33) mean value for malic acid hexoside 2.71 was observed in population 3 (Dareh‐Nezh; West Azerbaijan) and population 5 (Vinagh; East Azerbaijan. For malic acid hexoside 6.11, the highest (1,444.13) and lowest (210.92) mean value was observed in population 2 (Dareh‐Khan; West Azerbaijan) and population 4 (Aghberaz; East Azerbaijan). The highest (500.37) and lowest (134.08) mean value for coumaric acid was observed in population 2 (Dareh‐Khan; West Azerbaijan) and population 5 (Vinagh; East Azerbaijan). The highest (139.44) and lowest (92.26) mean value for coumaric acid 8.9 was observed in population 5 (Dareh‐Khan; West Azerbaijan) and population 4 (Aghberaz; East Azerbaijan). Concerning caftaric acid, the highest (11.56) and lowest (4.85) mean value was observed in population 2 (Dareh‐Khan; West Azerbaijan) and population 4 (Aghberaz; East Azerbaijan). The highest (7.58) and least (3.17) mean value for linoleic acid was found in population No. 5 (Vinagh; East Azerbaijan) and 1 (Kachleh; West Azerbaijan), respectively. The highest (161.84) and lowest (37.43) mean value for linoleic acid 5 was found in population 3 (Dareh‐Nezh; West Azerbaijan) and 1 (Kachleh; West Azerbaijan), respectively. About ellagic acid 11.49, the highest (3,040.49) and lowest (663.13) mean value was observed in population 5 (Vinagh; East Azerbaijan) and population 2 (Dareh‐Khan; West Azerbaijan). The highest (913.82) and lowest (495.18) mean value for ellagic acid 13.97 was observed in population 3 (Dareh‐Nezh; West Azerbaijan) and population 5 (Vinagh; East Azerbaijan), respectively.

**TABLE 2:** The values of descriptive statistics of phytochemical characteristics in studied sumac populations

Characteristic	Within population										Between population					
	X̅					CV										
	Pop1	Pop2	Pop3	Pop4	Pop5	Pop1	Pop2	Pop3	Pop4	Pop5	X̅	CVg	CVe	Vg	Ve	h^2^
Gallic acid 6.5	448.77	355.52	517.48	708.01	484.62	133.58	83.45	79.04	135.22	115.91	503.61	94.51	120.70	226,538	596,045.1	0.38
Gallic acid 8.7	553.88	887.00	806.71	719.2	532.54	149.49	75.1	85.6	109.44	101.77	702.13	80.45	101.41	319,106.4	826,054.7	0.39
Quercetin	92.29	64.09	71.33	42.82	46.18	129.18	140.71	75.72	148.83	132.19	62.38	102.19	126.53	4,062.848	10,292.38	0.39
Malic acid	29,799.5	77,321.64	60,891.27	10,712.1	7,393.5	113.91	81.76	60.62	90.1	80.6	38,040.87	308.35	97.16	13,758,654,198	15,124,751,259	0.91
Malic acid hexoside2.71	1659.86	1927.89	1940.35	749.46	525.33	129.58	82.23	43.35	115.77	81.17	1,407.32	159.33	91.91	5,027,990	6,700,885	0.75
Malic acid hexoside6.11	697.69	1,444.13	1,118.26	210.92	335.51	132.04	127.48	67.26	44.33	‐	1,041.47	110.56	122.40	1,325,882	2,951,022.69	0.45
Coumaric acid	334.03	500.37	377.76	144.05	134.08	118.77	60.08	79.06	52.8	102.54	298.06	203.28	90.23	367,119.4	439,452.7	0.84
Coumaric acid8.9	67.62	108.06	92.71	92.26	139.44	146.54	61.23	91.23	93.71	73.52	100.39	90.43	87.75	8,241.962	16,003.74	0.52
Caftaric acid	6.00	11.56	10.88	4.85	5.33	139.65	121.34	128.3	104.27	78.54	7.97	143.96	130.46	131.6408	239.75	0.55
Linoleic acid	3.17	6.44	6.63	3.98	7.58	128.22	61.39	59.9	102.02	93.5	5.54	115.52	84.92	40.89966	63.003	0.65
Linoleic acid5	37.43	89.04	161.84	80.93	142.96	122.55	95.57	134.97	126.25	132.78	105.06	167.69	140.06	31,039.78	52,691.5	0.59
Ellagic acid11.49	765.25	663.13	1,062.04	1526.2	3,040.49	121.67	72.94	105.81	115.12	79.23	1,446.71	254.12	105.35	13,516,162	15,839,104	0.85
Ellagic acid13.97	700.88	814.57	913.82	614.89	495.18	129.47	112.6	73.47	118.14	126.73	709.43	83.14	109.53	347,849	951,691.9	0.37

Abbreviation: X̅, mean value; CV, coefficient of variation; CVg, genotypic coefficient of variation; CVe, environmental coefficient of variation; Vg, genotypic variance; Ve, environmental variance; h^2^, heritability.

The highest (135.22) and least (79.04) coefficient of variation (CV) for gallic acid 6.5 was observed in population 4 and population 3, respectively. Concerning gallic acid 8.7, the highest (149.49) and lowest (75.1) CV was seen in population 1 and population 2. The highest (148.83) and lowest (75.72) CV for quercetin was seen in population 4 and 3, respectively. The highest (113.91) and least (60.62) CV for malic acid was observed in population 1 and population 3. The highest (129.58) and lowest (43.35) CV for malic acid hexoside 2.71 was found in populations 1 and 3. The highest (132.04) and lowest (44.33) CV for malic acid hexoside 6.11 was observed in population 1 and population 4. About coumaric acid, the highest (118.77) and lowest (52.8) CV was observed in population 1 and population 4. The highest (146.54) and lowest (61.23) CV for coumaric acid 8.9 was found in population 1 and population 2. For caftaric acid, the highest (139.65) and the least (78.54) CV was observed in population 1 and population 5. The highest (128.22) and lowest (59.9) CV for linoleic acid was observed in populations 1 and 3, respectively. The highest (134.97) and lowest (95.57) CV for linoleic acid 5 was observed in populations 3 and 2, respectively. The highest (121.67) and lowest (72.94) CV for ellagic acid 11.49 was seen in population 1 and population 2, respectively. Concerning ellagic acid 13.97, the highest (129.47) and lowest (73.47) CV was observed in population 1 and population 3, respectively (Table 2).

In comparing five studied sumac populations, the highest (38,040.87) and lowest (5.54) mean values were observed in malic acid and linoleic acid, respectively. Malic acid and gallic acid 8.7 had the highest (308.35) and lowest (80.45) genotypic coefficient of variation (CVg) among the five studied sumac populations. Malic acid and gallagic acid 13.97 showed the highest (0.91) and lowest (0.37) heritability among the five studied sumac populations (Table 2).

**TABLE** **2** The values of descriptive statistics of phytochemical characteristics in studied sumac populations.

### 3.2 Population structure

**FIGURE 3:**
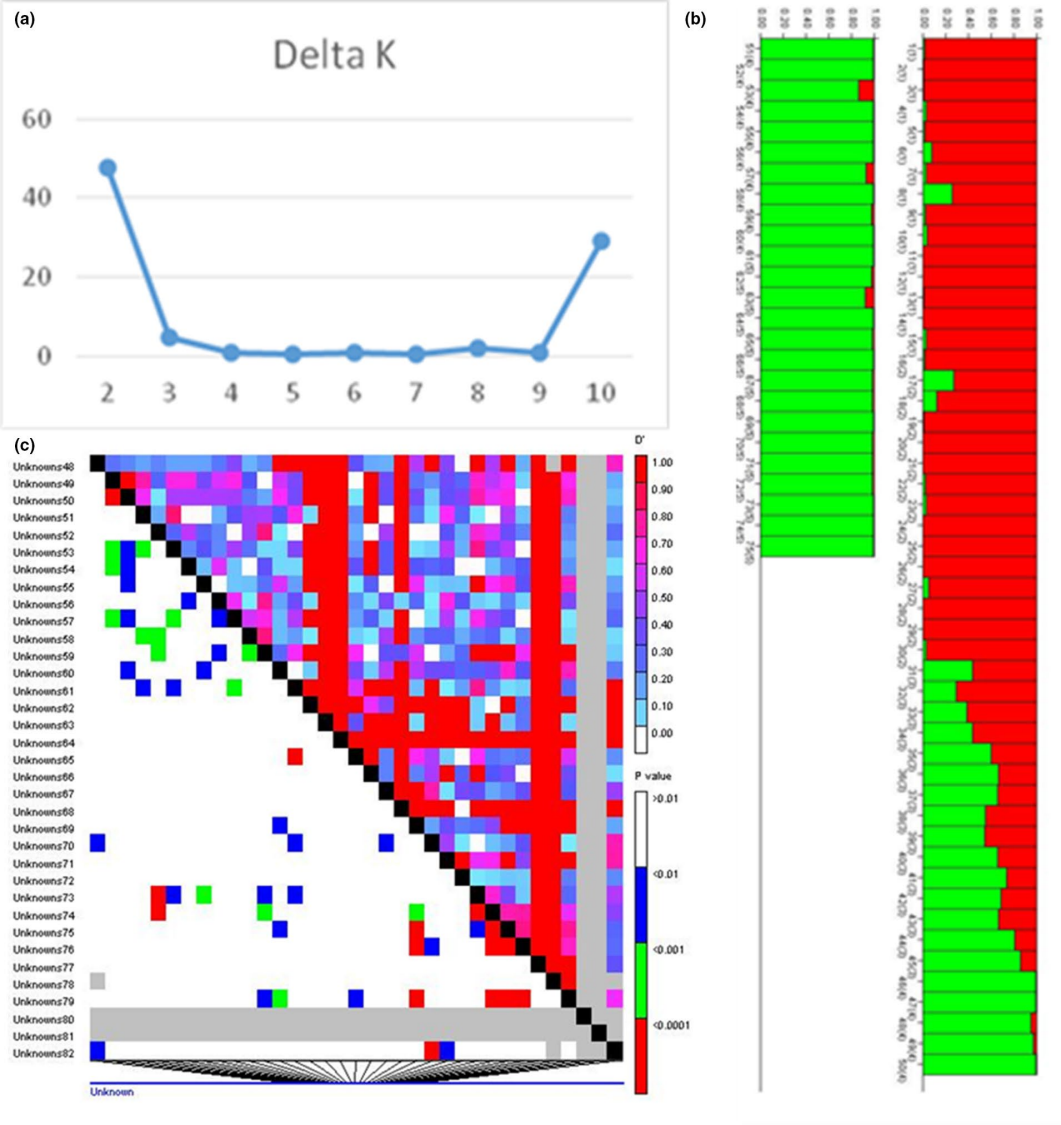
Model‐based Bayesian clustering among genotypes of sumac characterized using 132 ISSR markers. A, Bilateral chart to determine the optimal number of K identified by Structure program. B, Population structure indicating the two groups identified with different color bars. C, LD plot generated by ISSR marker pairs in 75 Iranian sumac (*Rhus coriaria* L.) genotypes. The upper diagonal shows D′ among each pair of markers. The lower diagonal shows the levels of significance between each pair of markers

The population structure in the sumacs (*Rhus coriaria* L.) association panel comprising 75 individuals was analyzed using 132 ISSR markers amplified by 18 ISSR primers. The optimal K value was 2, based on diagrams of LnP(D) and ΔK (Figure 3a). Structure analysis revealed that 63 individuals presented a membership probability of Q ≥ 0.70, and they were assigned to one of the two subpopulations. Twelve individuals with Q < 0.70 were determined admixed (Figure 3b). Results showed that 31 genotypes (41.33%) were assigned to subpopulation 1 and 32 genotypes (42.67%) assigned to subpopulation 2.

### 3.3 Linkage disequilibrium (LD) assay

LD was measured by r^2^ and D′. The r^2^ values among ISSR markers pairs showed an average value of 0.057. D′ value ranged from 0.000 to 1 with an average value of 0.59. A significant level of LD was observed in 11.64% of the ISSR markers pairs (*p* ≤.01) in the studied Iranian sumac germplasm (Figure 3c).

### 3.4 Association analysis

MLM was used for identifying molecular markers associated with genomic regions controlling phytochemical traits in the Iranian sumac association panel. Results showed a significant association (*p* ≤.05) of 12 ISSR markers with genomic regions controlling the studied traits (Table 4). Two QTLs (UBC856, UBC841) were identified for ellagic acid 13.97, one QTL (UBC856,) for quercetin, two QTLs (UBC857, UBC867) for caftaric acid, one QTL (UBC867) for linoleic acid, one QTL (UBC841) for gallic acid, two QTLs (UBC841, UBC842) for coumaric acid, two QTLs (UBC842, UBC826) for maleic acid hexoside 6.11, and one QTL (UBC823) for maleic acid (Table 3). Some markers were common for some traits. UBC856 was found to be associated with two characters (ellagic acid 13.97 and quercetin), UBC841 was found to be related with ellagic acid, gallic acid, and coumaric acid, UBC867 was found to be related with caftaric acid and linoleic acid, and UBC842 was found to be associated with coumaric acid and maleic acid hexoside 6.11 (Table 3).

**TABLE 3:** ISSR loci identified for the studied phytochemical traits in the studied sumac germplasm using association analysis

Characteristic	Locus	P value‐marker	*F* value‐marker	R^2^
Ellagic acid 13.97	UBC856	0.0015	10.95	0.17
	UBC841	0.022	5.53	0.10
Quercetin	UBC856	0.015	6.30	0.10
Caftaric acid	UBC857	0.0018	10.57	0.19
	UBC867	0.012	6.73	0.11
Linoleic acid5	UBC867	0.0021	10.31	0.15
Gallic acid8.7	UBC841	0.03	5.22	0.10
Coumaric acid	UBC841	0.027	5.14	0.11
Coumaric acid8.9	UBC842	0.014	6.33	0.097
Maleic acid hexoside 6.11	UBC842	0.0055	8.26	0.096
	UBC826	0.0021	10.31	0.12
Maleic acid	UBC823	0.0044	8.67	0.12

**TABLE** **3** ISSR loci identified for the studied phytochemical traits in the studied sumac germplasm using association mapping.

## 4 DISCUSSION

Results showed extensive phenotypic diversity within and between Iranian sumac populations. High phenotypic and genetic diversity is necessary for conducting association genetic. In the investigated Iranian sumac populations, malic acid was the predominant component and linoleic acid was a component with the lowest mean amount. Kossah et al., (2009) compared the chemical composition of Syrian and Chinese sumac, and they reported that the fruit of Syrian sumac contained higher amounts of organic acids than Chinese sumac and they observed that the predominant acid in both species was malic acid. It was reported that the major fatty acids in the sumac were oleic, linoleic, and palmitic acids (Dogan & Akgul, 2005; Ünver & Özcan, 2010).

According to Stansfield (1991), if the heritability of a trait is more than 0.5, the trait has high heritability; if it is between 0.2 and 0.5, the heritability is medium; and if it is lower than 0.2, it has low heritability. Given this, malic acid, malic acid hexoside 2.71, coumaric acid, coumaric acid 8.9, caftaric acid, linoleic acid, linoleic acid 5, and ellagic acid 11.49 showed high heritability. There are some reports about the low heritability of primary and secondary metabolites including sugars and organic acids in tomato fruits (Sauvage et al., 2014; Zhang et al., 2016).

A significant level of LD was observed in ISSR markers pairs in the studied Iranian sumac germplasm that encouraged us to run association genetic. LD is defined as a nonrandom association of alleles at different loci placed on the same or different chromosomes (linkage groups) (Mackay & Powell, 2007). A few studies presented reports about the LD patterns in crop plants (Khan & Korban, 2012; Yin et al., 2004). The structure of LD across the genome affects the resolution of association analysis (Remington et al., 2001). LD pattern is different between forest trees and fruit crops; domesticated trees have extended LD compared to undomesticated ones (Khan & Korban, 2012). Extension of LD between 50 and 100 cM in local Michigan populations of Arabidopsis was reported by Abdurakhmonov and Abdukarimov (2008). LD analysis in peach has shown increasing LD to 13–15 centimorgan (cM) (Aranzana et al., 2010). Genome‐wide LD in forest trees investigated and the results showed that LD in *Populus trichocarpa* extended up to 16–34 kb (Yin et al., 2004), less than 500 bp in *Populus termula* (Ingvarsson, 2005), 2000 bp in *Pinus taeda* (Brown et al., 2004), 1,000 bp in *Pseudostuga menziensii* (Krutovsky & Neale, 2005), and 100–200 bp in *Picea abies* (Rafalski & Morgante, 2004).

Based on structure analysis, the studied Iranian sumac association panel was subdivided into 2 subpopulations. The admixture of populations is an important factor that affects the power and precision of association genetic (Zhang et al., 2012). False‐positive marker–trait association occurs when the impact of population structure or familial relatedness was disregard (Yu et al., 2006). A mixed linear model can help scientists to overcome this problem by considering the troublesome agents as covariates in the model (Yu et al., 2006) and also increase resolution from the level of a 20 cM region to that of an individual gene (Sorkheh et al., 2008).

The mixed linear model was applied for detecting ISSR markers linked to genomic regions controlling phytochemical traits in the Iranian sumac association panel, and some significantly molecular markers were identified. Association studies in sumac have been used for identifying genomic regions related to fatty acid compositions (Qu et al., 2017; Zhu et al., 2019). It has been reported that the *FAD2* gene proceeds the linolenic acid synthesis (Zhu et al., 2019). Metabolite‐based association study has validated the metabolome GWAS in genetic improvement of complex traits (Matsuda et al., 2015; Wen et al., 2014). Schauer et al., (2008) detected up to 332 QTLs for the main tomato primary metabolites in a tomato IL population. Also, Sauvage et al., (2014) found 36 metabolite traits in a collection of tomato accessions by the GWAS approach.

Some association studies have been carried out in forest trees. For instance, Thumma et al., (2005) reported an association between a polymorphism in the *ccr* gene and microfibril angle in Eucalyptus. In another study, an association of SSR markers with resin yield was investigated in 240 genotypes of *Pinus roxburghii* and the results showed that two chloroplastic SSRs; Pt71936 and Pt87568 and one nuclear SSR; pm09a showed significant association with resin yield (Rawat et al., 2014). In maritime pine, Lepoittevin et al., (2012) found the association of 184 SNPs marker with the height growth rate. Also, Cabezas et al., (2015) found four SNPs associated with total height and polycyclism in maritime pines. Jugran et al., (2012) reported three ISSR markers associated with antioxidant activity in *Valeriana jatamansi*.

In the present study, some marker loci were common between traits. The common markers can be due to linkage or pleiotropic effects. The common markers are useful because it makes the possible simultaneous selection for several traits and can increase the efficiency of marker‐assisted selection programs.

## 5 CONCLUSIONS

Results of the present study suggest that the studied sumac germplasm was subdivided into 2 subpopulations. 84% of studied genotypes were considered to one of the two subpopulations and just 16% of them were assigned admixed. In the present study, QTLs controlling phytochemical traits in sumac were identified for the first time. A total of 12 ISSR markers associated with studied traits were identified using an association mapping approach. These ISSR markers provide primary molecular information for marker‐assisted selection in sumac. Results of MLM association mapping showed that some locus was common for more than one trait. These common markers are useful in sumac breeding programs and help in marker‐assisted selection programs.

## AUTHOR CONTRIBUTION

Data curation, formal analysis, investigation: [Rasoul Mohammadi Alaghuz]; conceptualization, methodology, supervision: [Reza Darvishzadeh]; resources, supervision: [Ahmad Alijanpour]; writing – original draft [Mitra Razi]; writing – review & editing: [Reza Darvishzadeh].

## CONFLICT OF INTEREST

The authors declare that they have no competing interests.

## Data Availability

The datasets generated during and/or analyzed during the current study are available from the corresponding author on reasonable request.
